# Efficacy of companion-integrated childbirth preparation for childbirth fear, self-efficacy, and maternal support in primigravid women in Malawi

**DOI:** 10.1186/s12884-019-2717-5

**Published:** 2020-01-21

**Authors:** Berlington M. J. Munkhondya, Tiwonge Ethel Munkhondya, Ellen Chirwa, Honghong Wang

**Affiliations:** 1 Xiangya School of Nursing of Central South University, 172 Tong Zi Po Road, Changsha, 410013 Hunan China; 2 Faculty of Midwifery, Neonatal and Reproductive Health Studies, Kamuzu College of Nursing, University of Malawi, P/Bag 1, Lilongwe, Malawi

**Keywords:** Primigravid women, Childbirth fear, Childbirth self-efficacy, Companion-integrated childbirth preparation, Pregnancy outcome, Maternity waiting homes

## Abstract

**Background:**

In resource-limited settings, childbirth remains a matter of life and death. High levels of childbirth fear in primigravid women are inevitable. To date, few studies have explored interventions to reduce childbirth fear in primigravid women. This study aimed to evaluate the efficacy of companion-integrated childbirth preparation (C-ICP) during late pregnancy for reducing childbirth fear and improving childbirth self-efficacy, birth companion support, and other selected pregnancy outcomes in primigravid women.

**Methods:**

A quasi-experimental study was carried out using a non-equivalent control group design to recruit a sample of 70 primigravid women in hospital maternity waiting homes in the intervention and control groups, with 35 in each group. The primigravid women and their birth companions in the intervention group received two sessions of companion-integrated childbirth preparation, whereas the control group received routine care. A questionnaire that incorporated the childbirth attitude questionnaire (CAQ), the childbirth self-efficacy inventory (CBSEI), the birth companion support questionnaire (BCSQ), and a review checklist of selected pregnancy outcomes was used to collect data. Pretest and post-test data were analyzed using simple linear regression. Beta coefficients were adjusted at a 95% confidence interval with statistical significance set at a *P*-value of < 0.05 using Statistical Package for the Social Sciences version 25.

**Results:**

At pretest, mean scores were similar in the intervention and control groups. At post-test, being in the intervention group significantly decreased childbirth fears (*β:* = − .866, *t* (68) = − 14.27, *p* < .001) and significantly increased childbirth self-efficacy (*β:* = .903, *t* (68) = 17.30, *p* < .001). In addition, being in the intervention group significantly increased birth companion support (*β*: = − 0.781, *t* (68) = 10.32, *p* < .001)*.* However, no statistically significant differences regarding pregnancy outcomes were observed between the study groups (Mann–Whiney U test, *p* > .05).

**Conclusion:**

The findings of our study suggest that C-ICP is a promising intervention to reduce childbirth fear while increasing childbirth self-efficacy and maternal support. We recommend the inclusion of C-ICP for primigravid women during late pregnancy in resource-limited settings.

## Background

Pregnancy and subsequent childbirth are perceived as a matter of life and death in resource limited-settings. Consequently, childbirth fear is inevitable in pregnant women as they embark on a perceived death-defying journey to motherhood [1]. According to a 2018 study in Malawi, more than 60% of pregnant women reported moderate to high childbirth fear; illiterate, unemployed, and young pregnant women were more likely to report such levels of childbirth fear [2]. The literature on perceptions and experiences of pregnancy has described a certain level of childbirth fear as a normal, protective psychological aspect of pregnancy and childbirth that may help a pregnant woman to prepare for and resolve challenging childbirth issues during pregnancy [3, 4].

Furthermore, Beiranvand et al. [5] reported that primigravid women were more prone to childbirth fear and may experience more adverse pregnancy outcomes than women who have given birth before. It is worth noting that childbirth fear levels are high in early and late pregnancy, suggesting that prompt support of primigravid women throughout pregnancy is vital to maintaining their psychosocial well-being and facilitating childbirth preparations [6]. However, the psychosocial well-being of pregnant women, including primigravid women, is overlooked and inadequately screened during antenatal care in resource-limited settings [7]. Care providers in resource-limited settings often not only overlook psychosocial elements during antenatal care but also fail to individualize psychosocial support. The failure of the health care providers to offer adequate preparation and psychosocial support results in vulnerable pregnant women, such as primigravid women, turning to alternative, traditional socio-cultural childbirth information. However, previous studies have reported that traditional childbirth information is not always helpful, and sometimes gives alarming information resulting in more childbirth fear among vulnerable primigravid women [7, 8].

On the other hand, results from previous studies suggest that the provision of active psychosocial and cultural support in terms of maternal childbirth information enhances maternal physiological processes, hope, and positive feelings that consequently result in a positive pregnancy in terms of childbirth self-efficacy and experiences [8, 9]. In this context, childbirth self-efficacy refers to beliefs and expectations that a pregnant woman has about childbirth [10]. Pregnant women with high levels of childbirth self-efficacy tend to internalize, master, and perform specific tasks that are expected of them during childbirth. Schwartz et al. [11] stated that childbirth self-efficacy is a psychosocial factor that can be modified through various efficacy-enhancing interventions. However, the lack of childbirth experience in primigravid women, coupled with cultural childbirth misconceptions [12], has a significant negative effect on childbirth self-efficacy [13].

The World Health Organization (WHO) standards recommend that every pregnant woman should receive psychological support to strengthen her capability of giving birth [10]. Kungwimba et al. [14] suggested a need for providing concrete physical, emotional, and informational support to primigravid women before giving birth. Furthermore, a randomized control trial that evaluated the effectiveness of receiving maternal social support from a female companion revealed that women who received the intervention were more satisfied with labor and delivery, and noted that satisfaction during labor was mainly associated with the presence of a birth companion [15].

Many developing countries have embraced the birth preparedness and complication readiness (BP/CR) strategy that identifies birth companions as a primary and crucial maternal support element enabling pregnant women to give birth at a health facility to mitigate high maternal mortality rates [16]. The BP/CR package during antenatal care equips pregnant women with a plan for the delivery location, transportation, birth companionship, blood donor, and items for a clean and safe childbirth [17]. The effectiveness of BP/CR is evident in the proportional increase of pregnant women who have given birth in health facilities in Malawi from 55% in 1992 to 91% in 2016 [18]. Therefore, birth companionship in resource-limited settings, such as Malawi, presents a perfect opportunity to offer concrete childbirth preparation, strengthening understanding and practical support for primigravid women to ascertain their positive childbirth experience [19].

Nevertheless, studies have reported issues with the misuse of birth companions who mostly provide instrumental support to care providers [20] and underutilization [21] in resource-limited settings. Birth companions rarely accompany a pregnant woman to the labor ward during childbirth but are sometimes allowed into the labor ward to intimidate and force the pregnant woman to cooperate with care providers’ demands [22]. Furthermore, Banda et al. reported in their study that 39% of birth companions unwillingly accompanied a pregnant woman to the hospital to give birth [19]. However, there is little documentation in the literature of studies that explored birth preparation intervention and examined the impact of birth companionship in resource-limited settings. Therefore, this study was designed to determine the efficacy of a structured childbirth preparation; companion-integrated childbirth preparation (C-ICP) package for primigravid women in Malawi.

### The conceptual framework for the study

**Fig. 1:**
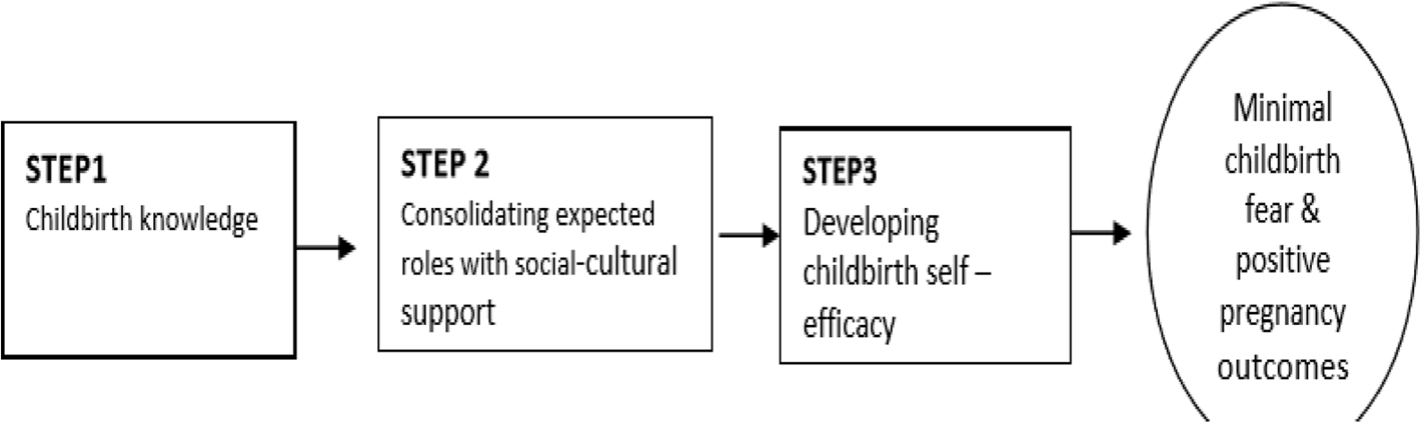
Companion-Integrated Childbirth Preparation (C-ICP) conceptual framework

The conceptual model used in this study was based on Meleis’s Transition Theory [23] that explains how providing support to an individual in a transition period may facilitate the development of resilience influenced by both an internal understanding of the challenging phenomenon and the external social support [5] (Fig. 1). This model is illustrated in this study using three steps of C-ICP on a primigravid woman to minimize childbirth fear and develop self-efficacy. The first step is the acquisition of childbirth knowledge, the second step consolidates relevant expected roles approved by her psychosocial and cultural environment, and the third step enables the development of self-efficacy in childbirth that minimizes childbirth fear, and that may, in turn, influence positive pregnancy outcomes in the primigravid woman. The current study intervention proposes that effective childbirth preparation should move from step one to three using dyadic interaction between a primigravid woman and her birth companion.

## Methods

### Study design

**Fig. 2:**
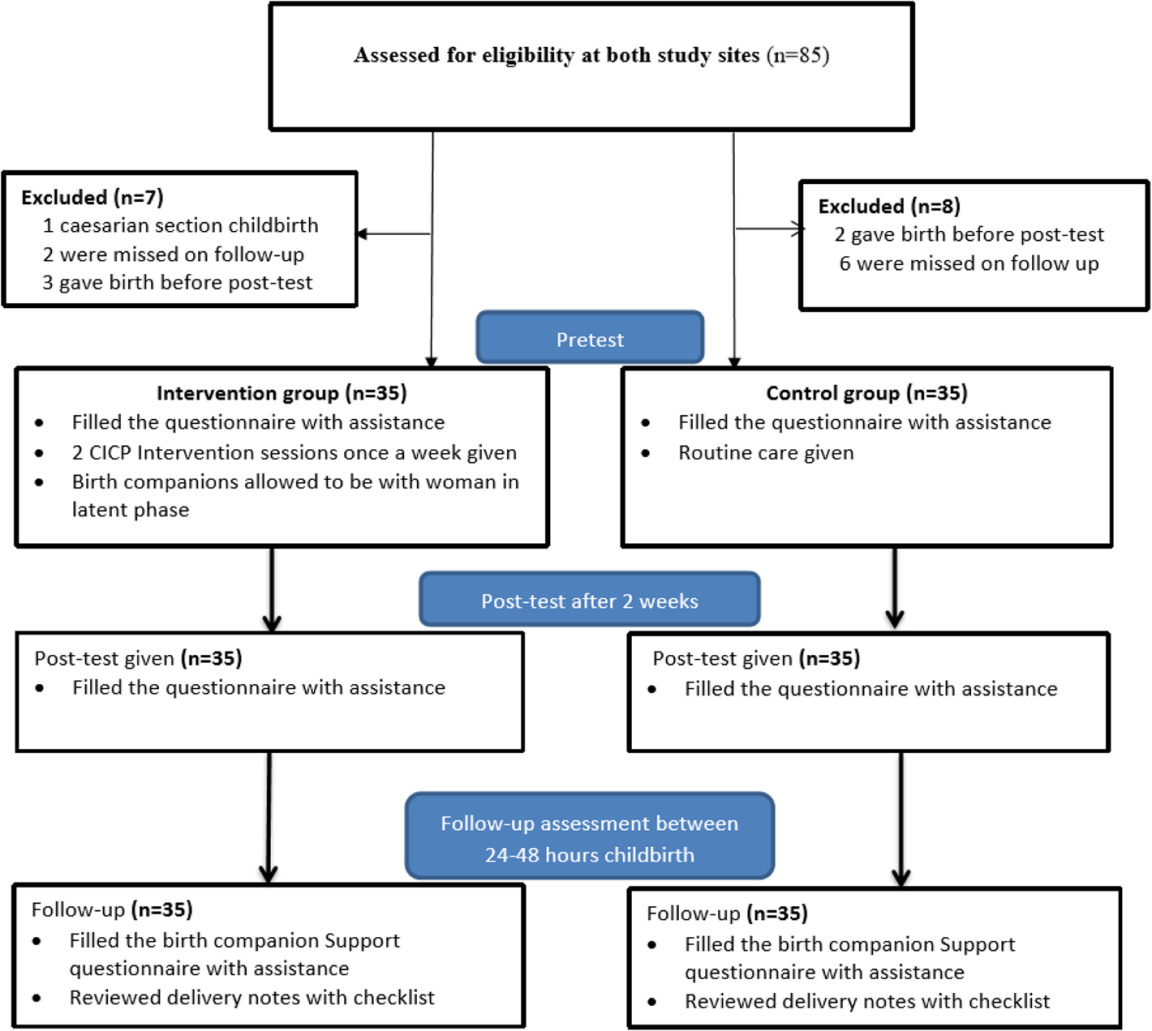
Flow profile of the study population: recruitment, allocation and measures

This was a pretest and post-test quasi-experimental study that used a non-equivalent control group to recruit primigravid women who were staying in hospital maternity waiting homes to await labor and give birth at the rural Mitundu and Kabudula community hospitals in Lilongwe, Malawi (Fig. 2). The baseline data were collected during the pretest study; post-test data were collected 2 weeks after the pretest, and the follow-up assessment was conducted between 24 and 48 h after childbirth. The quasi-experimental design was opted for instead of a randomized control trial to sidestep the increased risk of contamination in the control group. To achieve this, one health facility (Mitundu) was assigned as an intervention site, whereas the other facility acted as a control.

### Setting

Mitundu Community Hospital is situated in the outskirts of Lilongwe, Malawi’s capital, 38 km south of the city. It serves a population of about 127,000, with 6310 women giving birth per year, 2040 of which are primigravid women. Similarly, Kabudula community hospital is in the outskirts of Lilongwe, 57 km west of the city. It serves a population of about 43,014, including 4492 pregnant women, approximately 1800 of whom are primigravid women giving birth at the facility per year. These facilities were chosen because they offer a similar level of public health services in a rural population setting in Malawi, and they have maternity waiting homes. The maternity waiting homes are residential facilities located near the health facilities where pregnant women can wait for labour to give birth at a hospital [24]. Since we aimed to evaluate the efficacy of C-ICP during late pregnancy, we felt maternity waiting homes to be ideal settings in which to obtain our sample. In these facilities, pregnant women are admitted when they are between 35 and 40 weeks of gestation after attending required antenatal care visits [25]. Ideally, pregnant women would come to the maternity waiting homes during their last trimester [26], and they are always accompanied by a birth companion [20].

### Participants

To be recruited into the study, primigravid women needed to meet the following criteria: a) being a primigravid woman in late pregnancy with a singleton pregnancy, b) staying at the maternity waiting home while waiting for childbirth at a health facility, c) having a birth companion, and d) having the ability to communicate verbally in either Chichewa (the local language) or English. To be included in the study, the birth companion had to meet the following criteria: a) being a female birth companion accompanying the primigravid woman to the health facility for childbirth, b) staying at the hospital with the primigravid woman she has accompanied, and c) having the ability to communicate verbally in either Chichewa or English.

### Sample size

To guide the sample size calculation, we used the primary variable of childbirth fear. We reviewed previous experimental studies that used childbirth attitude scale. To our knowledge, no study was done in Africa that measured childbirth fear using childbirth attitude scale. However, in Iran, Nevaee and Abedian [27] study in an urban health care setting reported moderately large effect size (Cohen’s d = 0.58) while Khorsandi et al. [28] study at a specialized urban hospital setting reported very large effect size (Cohen’s d = 0.99). Furthermore, a study conducted in a rural community setting in India by Swaroopa & Deepthi [29] reported very large effect size (Cohen’s d = 1.25). In the current study, we expected large effect size (Cohen’s d = 0.80) on the reduction of childbirth fear mean score between the two study groups. We, therefore, used G*Power version 3.0.10 [30] with a priori power analysis for independent t-test on the following parameters: effect size of 0.8; alpha (α error prob) of 0.05; power (1- β err prob) of 0.95; and allocation ratio (N1/N2) of 1. The required sample size of 35 participants in each study group was reached (Fig. 2).

### Implementation of the C-ICP intervention strategy

The C-ICP intervention package was designed to educate and support the primigravid women and their birth companions in late pregnancy through structured childbirth education. The C-ICP package was adapted from *the BP/CR matrix* [31] and *the Integrated Maternal and Neonatal Health Practice guide* [32]. The C-ICP intervention builds on BP/CR elements, such as, birth companionship, danger signs and signs of labor, principles of woman-friendly care strategies effective for minimizing labor pains, and bearing down during childbirth, that may increase primigravid women’s confidence in giving birth [32].

Before implementation, the intervention’s applicability was enhanced through the rigorous consultative process with leading professional nurses and midwives at the selected health facilities to improve the reliability and acceptability of the package. Then, four research assistants (one registered nurse and three nurse technicians) at the intervention site and three (one registered nurse and two nurse technicians) at the control site were hired and trained to implement the C-ICP package in line with this study. The intervention was implemented using two educational sessions delivered to groups with a maximum of six pairs of participants each, one session per week for 2 weeks, upon their recruitment.

The C-ICP sessions were conducted at the antenatal clinic in the late afternoon hours after the clinic was closed. Each C-ICP session took approximately 1 h and 20 min and was delivered in Chichewa by at least two research assistants. Appropriate demonstrations and role-play of practical tasks were carried out during the sessions (Table 1). Each dyad was encouraged to interact to prepare for childbirth, and if they needed more support on childbirth preparation, they could seek help from facilitators or any care provider. Additionally, the arrangement was made with the hospital authorities to allow birth companions in the intervention group to accompany primigravid woman during the latent phase of labor at the waiting bay in the labor ward.

**Table 1:** Structured C-ICP courses for pregnant women and their companions

Topics	Content/Materials	Teaching Techniques
Required items and danger signs	Review the list of required items for clean childbirth and danger signs	Recall & Summarizing
Signs of labor	(a) regular, progressively painful contractions; (b) lower back pain radiating from fundus; (c) bloody show; (d) rupture of membranes or draining fluid	Recall & Summarizing
Effective pain reduction during labor and childbirth	(a) ambulation in early stage of labor; (b) relaxation and breathing techniques: start with one big breath, then take short and fast breaths; (c) between contractions: take 1–2 deep breaths, relax the body completely as the breath goes out; (d) back rub/massage; (e) frequent urination: every 2 h	Discussion/ Demonstration, Role-play, & Coaching
Effective bearing down during childbirth	(a) positioning: lithotomy (preferred); open legs, holding ankles with back curved and chin on chest; (b) start bearing down when asked to push and stop when contractions end; (c) effective pushing: pushing when feeling contractions; (d) relaxing and panting between contractions; (e) panting by opening mouth when asked during childbirth	A demonstration, Role-play, & Coaching
Expected roles of the pregnant woman when labor starts	(a) recognize normal signs of labor/danger signs and seek help fast; (b) eat adequately warm, soft porridge with enough sugar; (c) walk around in early-stage; (d) empty bladder every 2 h; (e) effective pushing when told	Discussion & Coaching
Expected roles of the birth companion	(a) do not give any local medicine/herbs; (b) stay/support when walking, and reassurance; (c) give back massages; (d) remind the pregnant woman of expected roles; (e) ensure pregnant woman receives assistance on time	Discussion & Coaching

### Measures and data collection

Trained research assistants conducted face-to-face interviews using an integrated questionnaire to collect data. The questionnaire consisted of five parts. The first part was used to collect the participant’s demographic characteristics including age, marital status, tribe, level of education, employment status of the woman and her partner, monthly income, and gestation age. The other four parts comprised of; the Childbirth Attitudes Questionnaire, the Childbirth Self-Efficacy Inventory (CBSEI), the Birth Companion Support Questionnaire (BCSQ) and Checklist for pregnancy outcomes (see an Additional file 1).

The CAQ was adopted to assess childbirth fear [33]. This tool was utilized for pretest and post-test measures in the present study. It was initially developed in 1981 by Areskog, Kjessler, and Uddenberg [34]. The version of the tool used in the study was a 16-item scale with a four-point Likert scale, having a total score range of 16 to 64 [33]. The higher scores represent high fear reported, with internal consistency reliability estimated at 0.83.

Childbirth self-efficacy was measured using the CBSEI part II [35], based on Bandura’s self-efficacy theory, which is used to measure a pregnant woman’s ability to cope with labor and childbirth [33]. The short version was developed [36] with 32 items, each of the two subscales containing 16 items on a Likert scale of 1 to 10. The Arabic translation of the CBSEI has demonstrated a high level of internal consistency, achieving 0.86 for the total outcome expectancy subscale and 0.90 for the total self-efficacy expectancy subscale [37]. This tool was previously piloted in a neighboring country, Tanzania, where a highly reliable internal consistency with a Cronbach’s alpha of 0.80 was indicated, suggesting that the questions were well understood by pregnant women in the Tanzanian culture [38]. Therefore, this result has demonstrated that the CBSEI tool can be used in a different cultural setting from the one in which it was originally developed. Although the tool has not been validated in Malawi, it was chosen because it had previously proven to be useful when applied either in the late third trimester or before the impending birth.

Birth companion support was measured using the BCSQ [39]. The tool was developed to measure women’s perceptions of emotional and tangible aspects of functional support provided by the support person during labor and birth [39]. In the present study, this tool was used at the pretest assessment and the follow-up assessment between 24 and 48 h after the participant had given birth. The BCSQ was modified from the Labor Support Questionnaire [40] by Dunne et al. [39]. The BCSQ contains 13 items designed to measure emotional and tangible support with a four-point Likert ordinal response of 0 (not at all), 1 (a little), 2 (most of the time), and 3 (all the time). It has a reported Cronbach’s alpha of 0.80 [38].

The CAQ, CBSEI, and BCSQ were originally written in English, and we adapted and translated them into Chichewa by a bilingual translator (forward translation) with the authors’ permission to use the tools. The rigorous consultative process enhanced the applicability for professional nurses and midwives delivering care in the targeted rural community hospitals. The translated versions were then checked and discussed for wording and clarity by four midwives providing care to women at the antenatal clinic and labor ward. The researcher who has a midwifery background facilitated the process to ensure that the translated instrument versions retained core elements that the instruments were supposed to measure. Finally, the Chichewa versions were translated back into English by an independent bilingual translator (back-translation). The original and back-translated questionnaires were discussed separately and compared for clarity and inconsistencies by nurses and midwives at Mitundu and Kabudula community hospitals before reaching a consensus on the final version. To our knowledge, no study in Malawi has validated these tools. However, a pilot study was conducted to assess the applicability and clarity of the translated Chichewa versions, and modifications were made accordingly. The participants in the pilot study were15 primigravid women staying at a health center facility while waiting to give birth in Lilongwe, Malawi. We noted that primigravid women were more conversant with the Likert scale when they were asked to consider it from the perspective of their familiar practices at the maize mill, where a calibrated stick is used to determine the price on the of maize they want to process. This understanding helped the participants to give appropriate responses. The CAQ and CBSEI tools were also used in our survey on childbirth fear (being considered for publication), with good responses from the participants indicating a Cronbach’s alpha of 0.86 for the CAQ and 0.83 for the CBSEI, whereas the pilot study indicated that the BCSQ’s Cronbach’s alpha was 0.76.

A checklist review was conducted to collect data on the selected pregnancy outcomes. The researcher reviewed delivery notes using the checklist between 24 to 48 h after childbirth to capture the information on pregnancy outcomes, including gestation age, observed danger signs, problems experienced, duration of the first, second, and third stages of labor, type of delivery, perineal trauma, Apgar score, and the time when exclusive breastfeeding was initiated.

### Ethical considerations

This study was approved by the Institutional Review Board of the Xiangya School of Nursing, Central South University, and The National Committee on Research in the Social Sciences and Humanities in Malawi (REF.NO.NCST/RTT/2/6). Participants, including birth companions in the intervention arm, received oral information about the study’s risks and benefits, that their participation was voluntary, that they could withdraw at any point without any reprisal, and that their names would remain confidential. Written informed consent was obtained from each participant and her birth companion. Oral consent from legal guardians was also obtained for those pregnant women under 18 years old.

### Data analysis

The Statistical Package for the Social Sciences (SPSS) version 25 (IBM corp., Armonk, NY, USA) was used for data analysis. An independent t-test was used for continuous variables to compare the baseline data and posttest measures between the study groups. Mann–Whitney U tests were used to compare the two groups’ demographic characteristics and selected pregnancy outcomes in follow-up assessments due to a lack of normality, and *χ*^2^ tests were used for categorical variables. Simple linear regression was used to determine if the C-ICP intervention was significantly more efficacious at decreasing childbirth fear while increasing self-efficacy and maternal support than routine care. A statistical significance level was set at *p* < .05. The beta coefficients were reported adjusted at a 95% confidence interval [10].

## Results

### Participants’ characteristics

Data were collected from February 2018 to August 2018. In total, 70 primigravid women were recruited, with 35 into the intervention group at Mitundu Community Hospital and 35 in the control group at Kabudula Community Hospital (Fig. 2). A descriptive analysis was performed for the baseline demographic characteristics. The mean age was 19.97, with standard deviation 2.71 and the age range of 16 to 31 years. All participants were from the Chewa tribe, and the majority was married. As shown in Table 2, there were no statistically significant differences in demographic characteristics between the intervention and control groups.

**Table 2:** Demographics of participants in the intervention and control groups

Variables	Intervention group (*n* = 35)	Control group (*n* = 35)	Test		*P*-value
			*t*	*χ*^2^	
Age (years) mean ± SD	19.83 ± 2.90	20.11 ± 2.70	0.429		0.669
Marital Status					
Single	4 (11.4%)	7 (20%)			
Married	31 (88.6%)	28 (80%)	N/A	0.981	0.513
Education Level					
None	2 (5.7%)	0 (0%)			
Primary	20 (57.1%)	23 (65.7%)			
Secondary or above	13 (37.1%)	12 (34.3%)	N/A	3.022	0.461
Occupation					
Housewife/farmer	32 (91.4%)	28 (80.0%)			
Businesswoman	2 (5.7%)	5 (14.3%)			
Employee	1 (2.9%)	2 (5.7%)	N/A	1.935	0.430
Partner’s Occupation					
Farmer	29 (82.9%)	26 (74.3%)			
Businessman	2 (5.7%)	5 (14.3%)			
Employee	2 (5.7%)	1 (2.9%)	N/A		
None	2 (5.7%)	3 (8.6%)		2.033	0.678
Income per Month ($USD)					
< 13	21 (60.0%)	15 (42.9%)			
13–26	12 (34.3%)	18 (5.4%)			
> 26	2 (5.7%)	2 (5.7%)	N/A	2.213	0.359

*SD*, Standard Deviation; *t*, t-test; *χ*^*2*^ = chi-squared test; *N/A*, Not applicable; *$USD*, United States Dollar ($1 USD ≈ 750 Malawi Kwacha)

### Differences between groups regarding childbirth fear, childbirth self-efficacy, and birth companion support

The independent samples t-test indicated that there was no statistically significant difference in the average pretest scores for childbirth fear (*t* (68) = .783, *p* = .436), childbirth self**-**efficacy (*t* (68) = − 1.20, *p* = .234), and birth companion support (*t* (68) = − 1.66, *p* = .102) between the intervention and the control groups (Table 3). Additionally, the post-test measures of childbirth fear, childbirth self-efficacy, and birth companion support between the study groups were assessed and assumed to be normally distributed [41]. Therefore, we opted for a linear regression approach to analyze the efficacy of the C-ICP intervention for childbirth fear, childbirth self-efficacy and birth companion support on post-test measures in the present study.

**Table 3:** Intervention and control group mean score differences

Variable	Group				95% CI means difference		*P*-value
	Intervention		Control				
	Mean	SD	Mean	SD	Lower	Upper	
Fear of childbirth							
Pretest	40.29	6.78	39.11	5.67	− 1.175	5.346	0.436
Post-test	23.54	3.60	40.77	4.31	− 18.668	− 14.075	0.001
Childbirth self-efficacy							
Pretest	199.37	34.02	208.26	27.50	−23.641	5.869	0.234
Post-test	301.43	11.80	208.37	29.55	82.222	103.892	0.001
Birth companion support							
Pretest	25.71	4.99	26.15	3.89	− 3.905	0.362	0.102
Follow-up	37.97	1.42	30.86	3.82	5.725	8.504	0.001

*SD*, Standard Deviation; *CI*, Confidence Interval

**Fig. 3:**
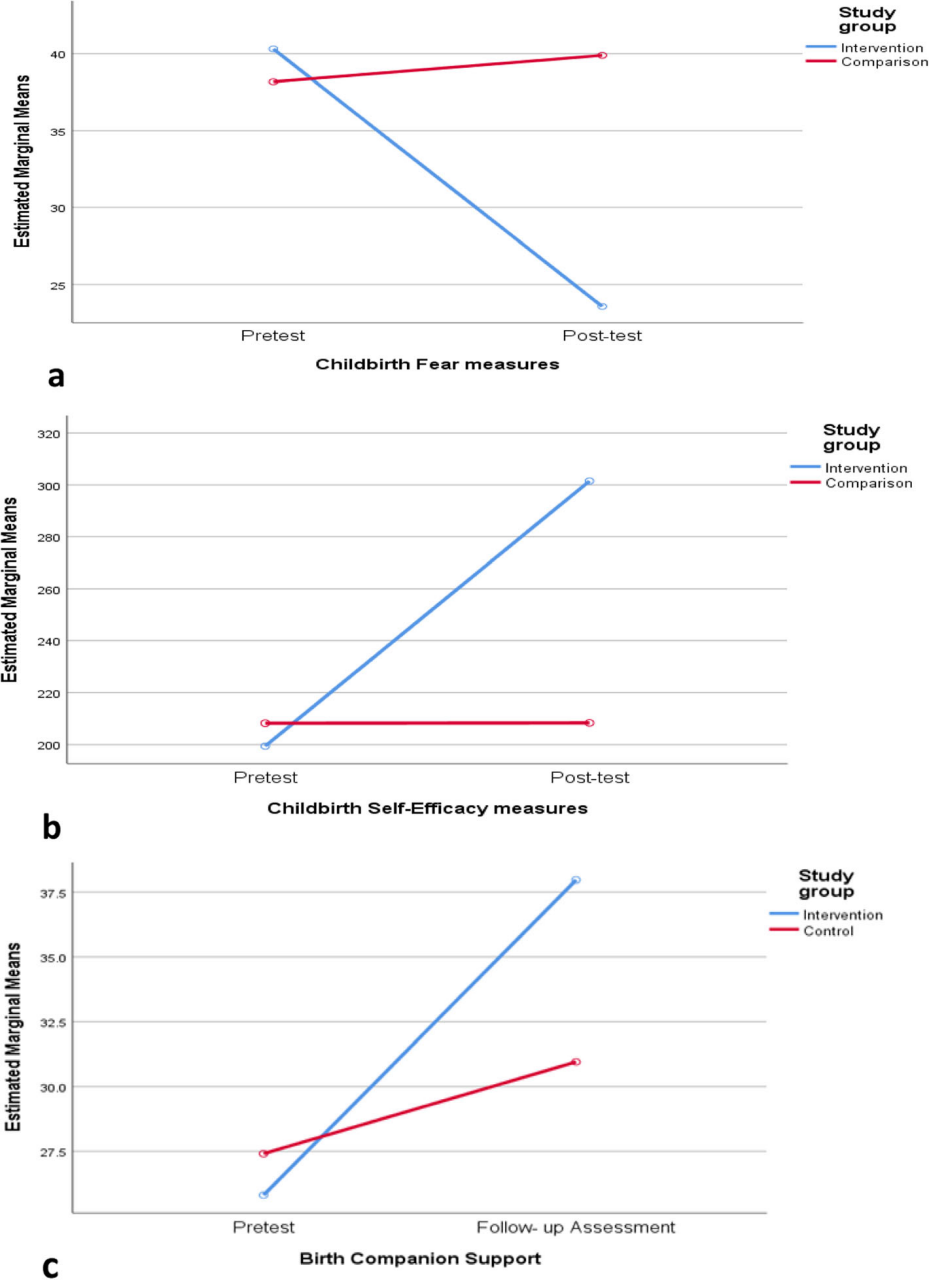
Line graphs comparing measures in the intervention and control groups. **a** Childbirth fear measure. **b** Childbirth self-efficacy measure. **c** Birth companion support measure

The childbirth fear result indicated a lower mean score in the C-ICP intervention group compared to the group that received routine care (Table 3). The simple linear regression indicated that the C-ICP intervention was significantly more efficacious than routine care for childbirth fear (*β:* = − .866, *t* (68) = − 14.27, *p* < .001) (see Fig. 3a). The C-ICP intervention explained a significant proportion of variance in childbirth fear scores (*R*^*2*^ = .75, *F* (1, 68) = 203.75, *p* < .001). In this sample, the level of education significantly predicted treatment effect (*p* < .001), whereas age was not significant (Table 4).

**Table 4:** Comparison of the intervention and control group measures using simple linear regression

Variable	Factors	(β)	*t*	95% CI		*R*^*2*^	*F (df)*	*P-value*
				Lower bound	Upper bound			
Childbirth Fear	Study Group	−0.866	−14.274	−18.66	−14.08	0.75	203.75(1,68)	0.001
	Age	−0.059	0.213	− 0.59	0.18		88.40(1,66)	0.295
	Education Level	0.228	4.089	2.09	6.08		88.40(1,66)	0.001
Childbirth Self-Efficacy	Study Group	0.903	17.303	9 8.35	132.28	0.82	299.38(1,68)	0.001
	Age	0.001	0.011	−1.99	2.01		97.96(3,66)	0.991
	Education Level	− 0.041	− 0.771	− 14.47	6.40		97.96(3,66)	0.443
Birth Companion Support	Study group (follow-up)	0.781	10.322	5.74	8.49	0.61	106.55(1,68)	0.001
	Age	−0.035	0.452	− 0.32	0.20		34.68(3,66)	0.653
	Education level	−0.006	0.077	− 1.39	1.29		34.68(3,66)	0.938

*β*, Standardized Coefficient Beta; *t*, t - statistic; *95 CI*, Confidence Interval; *R*^*2*^, R Square; *F*, *F*-Statistic; *df*, degrees of freedom

Similarly, the childbirth self-efficacy result indicated a high mean score in the group that received C-ICP intervention compared to the group that received routine care (Table 3). The linear regression indicated that the C-ICP intervention was significantly more effective for enhancing childbirth self-efficacy than routine care (*β:* = .903, *t* (68) = 17.30, *p* < .001) (see Fig.3b). The C-ICP intervention explained a significant proportion of variance in childbirth self-efficacy scores (*R*^2^ = .82, *F* (3, 68) = 299.38, *p* < .001). Age and education level were not statistically significant (*p* > .05) (Table 4).

The birth companion support on follow-up measure indicated a higher mean birth companion support score in the group that received C-ICP intervention compared to the group that received routine care (Table 3). The linear regression indicated that the C-ICP intervention was significantly more efficacious than routine care (*β*: = − 0.781, *t* (68) = 10.32, *p* < .001) (see Fig. 3c). The proportion of variance explained by C-ICP intervention was significant (*R*^*2*^ = 0.61, *F* (1,68) =106.55, *p* < .001), whereas age and education level were not statistically significant (*p* > .05) (Table 4).

### Differences in pregnancy outcomes

We observed that the length of the second stage of labor in the intervention group (mean = 25.29 min) was shorter than in the control group (mea*n* = 27.14 min), but the difference was not statistically significant (Table 5). There were no differences in other selected pregnancy outcomes between the intervention and control groups (Mann–Whiney U test, *p* > 0.05).

**Table 5:** Group comparison of selected pregnancy outcomes in follow-up assessment

Variables	Intervention (*n* = 35)	Control (*n* = 35)	Mann–Whitney U test	Pearson/ Fisher’s test	*P*-value
			U	χ2	
Gestation age > 38 weeks	35 (50%)	35 (50%)	N/A	N/A	N/A
Danger signs observed					
Yes	0 (0%)	2 (5.7%)			
No	35 (100%)	33 (93.4%)	N/A	2.831	0.920
Problems experienced					
Yes	0 (0%)	2 (5.7%)			
No	35 (100%)	33 (93.4%)	N/A	2.831	0.920
The first stage of labour	11.72^+^	12.44^+^	−1.23	N/A	0.224
The second stage of labour	25.29	27.14	−1.021	N/A	0.311
The third stage of labour	5.31	5.14	1.164	N/A	0.249
Perineal trauma					
Intact	17 (48.6%)	10 (28.6%)			
Laceration	11 (31.4%)	16 (45.7%)			
First-degree tear	7 (20%)	9 (25.7%)	N/A	3.018	0.285
Apgar score at 1 min					
Normal 7/10 to 10/10	35 (53.7%)	34 (49.3%)			
Moderate 4/10 to 6/10	0 (0%)	1 (2.9%)	N/A	3.446	0.237
Cooperation with providers’ instructions	35 (50%)	35 (50%)	N/A	N/A	N/A
Initiation of exclusive breastfeeding	35 (50%)	35 (50%)	N/A	N/A	N/A

^+^ missing data n = 27; *N/A*, Not applicable; *U*, Mann–Whiney U test; *χ*^*2*^, chi-squared test

## Discussion

The results of our study showed a significant reduction of childbirth fear, enhanced childbirth self-efficacy, and increased birth companion social support in the intervention group compared with the control group. The changes in childbirth fear, and childbirth self-efficacy during the post-intervention measures as well as birth companion support during the follow-up assessment were markedly significant in the present study. These findings agree with a similar study on a psycho-education program intervention that was reported to reduce childbirth fear in primigravid women [42]. It is evident from the present study’s findings that adequate childbirth preparation in primigravid women is vital to simultaneously improve their psychosocial well-being and enhancing their ability to give birth.

It was noted that most of the women attended the required four focused antenatal care visits before they were admitted to the maternity waiting homes to give birth. The high levels of childbirth fear in this sample at pretest reflect the inability of the current prenatal services to meet the childbirth preparation needs of primigravid women. Primigravid women may receive conflicting information on pregnancy and childbirth that may compromise their confidence in giving birth [43]. The ability of primigravid women to withstand the stress of giving birth during labour is exaggerated by not only lack of knowledge about childbirth, but also the misinformation they receive concerning pregnancy and childbirth from the social environment due to ineffective prenatal care [44]. Therefore, the need for more practical information in the social support context during late pregnancy is vital for helping primigravid women approach childbirth positively.

Our study’s findings also indicated that primigravid women who received C-ICP intervention had higher levels of self-efficacy than those who received routine care. The present study’s results are in line with findings by Azim et al. that a socio-culturally relevant support structure has positive effects on developing self-efficacy in pregnant women [45]. Our study has demonstrated that C-ICP is effective for primigravid women during late pregnancy. One reason is that the detailed and step-by-step pre-birth instructions helped the primigravid women to be familiar with the delivery process. Another reason is that the involvement of the birth companion might have helped the primigravid women to accept and consolidate childbirth information and to have positive perceptions of the childbearing phenomenon. The evident practical support of the birth companion during late pregnancy might have helped the primigravid women to work through their childbirth fears and develop childbirth confidence in line with their socio-cultural values and expectations. This finding is similar to the Senanayake et al. study that reported on the impact the presence of a birth companion from the mother’s social network had on providing support to the woman giving birth [46].

We anticipated minimal changes in terms of birth companion support between the study groups because, in Malawi, the female support person is a prerequisite element for hospital delivery and maternity waiting home admission at a birthing facility, in line with the BP/CR strategy [19]. Additionally, primigravid women could be less apprehensive and more ecstatic after childbirth, hence reporting positive birth companion support. In the current study, birth companion support revealed a greater change in the intervention group than in the control group. These results were desirable, as primigravid women are generally more apprehensive about childbirth and need concrete social support before and after childbirth [8]. This finding reflects on the importance of providing primigravid women with tailored socio-cultural support during prenatal care to enhance childbirth preparation. Our results were consistent with a systematic scoping review to identify the processes and outcomes of antenatal care provision that are important to the health of pregnant women [47]. We suggest that future studies should consider extending the follow-up assessment to a longer period to assess the impact of the intervention on future childbirth.

It is the expectation of our study design that C-ICP intervention will not only enhance the maternal social and cultural support but also have a ripple effect of empowering and influencing women in the society through this enhanced birth companionship during childbirth. Although the study did not further examine the acceptability of C-ICP intervention, the implementation experience and the enthusiasm of the participants in the intervention group throughout the study may have indicated a positive attitude toward the C-ICP intervention strategy. Furthermore, no participant opted out of this study nor complained about the study intervention.

Unexpectedly, our findings did not show significant differences between the intervention and control groups on the selected pregnancy outcomes. One reason for this finding was that pregnancy outcomes in this sample were good in general and left very little space for making a difference. Another reason was the lack of accuracy in documenting labor and childbirth events retrospectively, which remains a challenge in study designs that review medical notes in resource-limited contexts [48]. However, a study in Norway used maternal ward electronic birth records that reported a statistically significant longer duration of labor for women with childbirth fear compared to women without childbirth fear [49]. Future studies in resource-limited settings should deliberately be designed to measure labor stages and pregnancy outcomes precisely.

### Limitations of the study

This study had some limitations. First, its target population was primigravid women with normal pregnancy events in a rural hospital setting; thus, the results may not be generalizable to other hospital settings. Second, quasi-experimental design was used in facilities that have different population sizes and population proportions of pregnant women which could compromise the internal validity of the study. Third, the use of multiple statistical tests on a relatively small sample size might have increased interpretation errors of the study results. However, the effect sizes for the study outcomes were considerably large, suggesting that adjusting *p*-value for multiple tests would not have changed the results. Lastly, the follow-up assessment was done soon after childbirth; hence, we did not evaluate the long-term effects of C-ICP intervention. Therefore, a future randomized control trial with large sample size and a longer follow-up duration is recommended to determine the robustness of the C-ICP intervention.

### Implications for practices and health policies

Current prenatal care services in resource-limited settings, rarely engage with and fully utilize birth companions in preparation for childbirth because the birth companions lack good orientation and preparation to offer psychosocial support. The engagement of the primigravid woman and her birth companion with childbirth preparation will not only promote childbirth self-efficacy but also ensure quality prenatal care services to primigravid women in resource-limited settings. Furthermore, the empowerment of birth companions in terms of childbirth information may have a long-term positive impact on maternal social support in the community. Therefore, national policies governing prenatal care services in resource-limited settings should embrace C-ICP to guarantee positive childbirth experiences for primigravid women.

## Conclusion

Our study has demonstrated the efficacy of C-ICP at reducing childbirth fear and enhancing self-efficacy and maternal support in the late pregnancy period among primigravid women. The result suggests the positive role that birth companions may play in reducing childbirth fear by enhancing childbirth self-efficacy in primigravid women in resource-limited settings that can help to strengthen the BP/CR strategy. We suggest that C-ICP should be part of antenatal care services in late pregnancy to enhance childbirth preparation in primigravid women in addition to the current four antenatal care visits in routine antenatal care practice.

## Supplementary information

**Additional file 1.** Data Collection Instrument

## Data Availability

The datasets used and analyzed during the current study are available from the corresponding author upon request.

## Abbreviations

BCSQ: Birth Companion Support Questionnaire
BP/CR: Birth Preparedness and Complication Readiness
CAQ: Childbirth Attitude Questionnaire
CBSEI: Childbirth Self-Efficacy Inventory
C-ICP: Companion- Integrated Childbirth Preparation
NHSRC: National Health Science Research Committee
SPSS: The Statistical Package for the Social Sciences
WHO: World Health Organization
