# Supplementary material for: Efficacy of companion-integrated childbirth preparation for childbirth fear, self-efficacy, and maternal support in primigravid women in Malawi
Source: BMC Pregnancy Childbirth. 2020 Jan 21;20:48. doi: 10.1186/s12884-019-2717-5 (PMC6975021; doi:10.1186/s12884-019-2717-5)
Supplement: Supplementary file 1 — Additional file 1. Data Collection Instrument [file 12884_2019_2717_MOESM1_ESM.pdf]

Companion-Integrated Childbirth Preparation study questionnaire

Participant Study Code………………………….

Date……/……/……

Part 1:

Demographic Data

Please fill in the personal details and where necessary tick ☑ one answer for each question, unless otherwise specified.

1. Age: ________ (in years)
2. Marital status:
   - ☐ Married
   - ☐ Single
   - ☐ Separated
   - ☐ Divorced
   - ☐ Widowed
3. Tribe ____
4. Education Level:
   - ☐ Did not attend school
   - ☐ Primary Level
   - ☐ Secondary level
   - ☐ Tertiary level
5. Your occupation ______________________________
6. Your partner's occupation ______________________________
7. Estimated monthly income in Malawi Kwacha *(750 Malawi Kwacha≈$1 USD)*
   - ☐ <10,000
   - ☐ 10,000-20,000
   - ☐ >20,000-30,000

Part 2: Childbirth Attitude Questionnaire (Adapted from Lowe, 1991). This tool is only available upon request from the developer.

Part 3: Childbirth Self-Efficacy Inventory (CBSEI) (Adapted from Lowe, 1991). This tool is only available upon request from the developer.

Part 4: Birth Companion Support Questionnaire (Adapted from Dunne, Fraser, & Gardner, 2014). This tool is only available upon request from the developer.

Part 5. Checklist for pregnancy outcomes

Participant Study Code…………………………. Date…../……/……

Review medical records for labour and delivery between 24-48 hours after childbirth

No	Item	**Observed** (yes/no) or **Values**	Missing	Remarks
1	Gestational age			
2	Danger signs			
3	Problems experienced			
4	Duration of the first stage of labour			
5	Duration of the second stage of labour			
6	Duration of the third stage of labour			
7	Perineal trauma sustained			
	• Intact			
	• Laceration			
	• Degree of tear			
8	Apgar score at 1 minute			
	• Normal (7/10 to 10/10)			
	• Moderate (4/10 to 6/10)			
	• Severe (≤ 3/10)			
9	Apgar's Score baby after birth			
10	Client's cooperation with care			
11	Initiates breastfeeding within 30min after birth			
